# Temporomandibular disorders, voice and oral quality of life in women

**DOI:** 10.1590/S1678-77572009000700009

**Published:** 2009

**Authors:** Tatiane Cristina PEREIRA, Alcione Ghedini BRASOLOTTO, Paulo César CONTI, Giédre BERRETIN-FELIX

**Affiliations:** 1 MSc Student, Department of Speech-Language Pathology and Audiology, Bauru School of Dentistry, University of São Paulo, Bauru, SP, Brazil; 2 MSc, PhD, Assistant Professor, Department of Speech-Language Pathology and Audiology, Bauru School of Dentistry, University of São Paulo, Bauru, SP, Brazil; 3 DDS, PhD, Associate Professor, Department of Prosthodontics, Bauru School of Dentistry, University of São Paulo, Bauru, SP, Brazil

**Keywords:** Temporomandibular joint disorders, Voice, Quality of life

## Abstract

Some studies have shown a relationship between temporomandibular disorders (TMD) and dysphonia, as well as quality of life in oral health. Objective: The purpose of this study was to investigate the correlation between severity of vocal self-perception and TMD severity and the correlation between oral health-related quality of life impairment and TMD severity. Material and methods: Thirty-three women aged 20 to 40 years, with or without complaint of dysphonia, were recruited at the Bauru campus of the University of São Paulo, Brazil, and the local community. All participants were subjected to an investigation of quality of life related to dental and speech aspects by the application of Oral Health Impact Profile-short form (OHIP-14) and the Voice-Related Quality of Life (V-RQOL) protocol. Also, a questionnaire was applied to detect the presence and severity of TMD. Results: There was significant correlation between TMD and quality of life for all aspects analyzed in the oral health protocol, except for function and physical limitation (p>0.05). There was negative correlation between TMD and voice-related quality of life in the total score (p=0.007) as weel as physical (p=0.008) and socio-emotional aspects (p=0.017). In addition, there was statistically significant correlation between TMD and vocal self-perception (p=0.037). Conclusion: There is an association between TMD severity, voice-related and oral health-related quality of life. It is important to investigate in future studies the vocal self perception as well as the oral and voice conditions in patients with TMD.

## INTRODUCTION

Temporomandibular disorders (TMD) result from abnormal functioning of the masticatory muscles, temporomandibular joints (TMJs), associated structures or both^8^. TMD are considered as a multifactorial manifestation and can be related to parafunctional habits such as tooth clenching and/or bruxism, head or neck traumas, unstable bite, postural problems, and emotional stress, among others^24^.

Individuals with TMD may present headaches or neck pain, TMJ noises, tinnitus or ear fullness, crepitation^10^ on opening or closing the mouth, opening limitation, and difficulties in chewing^9^^,^^11^ and on the speech^5^^,^^6^. TMD can even influence individual’s psychosomatic characteristics reducing their quality of life^2^.

The Oral Health Impact Profile (OHIP) is a scaled index developed in Australia to measure oral health related to quality of life. This questionnaire has been used in its German version to characterize quality of life related to oral health in individuals with TMD, and scores of the questionnaire indicated damages in different categories of diagnosis, with greater emphasis on the psychosocial axis^16^. The OHIP has also been applied in Slovak patients, resulting that individuals with TMD have more physical symptoms related to injury, and greater commitment of the oral health-related quality of life with the increase of age^27^.

In addition to the TMD impact on orofacial functions and the individuals’ quality of life, vocal changes are signs and symptoms commonly associated with TMD cases. This occurs due to the fact that TMD etiologic factors are common to dysphonia, such as excessive tension in the cervix and orofacial region and mouth opening restriction, since mandibular movement limitation during speech can affect voice acoustics^27^.

In cases of dysphonia, quality of life and emotional condition are also compromised. In the last years, a number of scales have been developed^19^, making possible the individual self-assessment about the severity of one’s voice problem. The Voice-Related Quality of Life (V-RQOL) protocol assesses individual’s perception of the voice problem impact on one’s life^14^. The Brazilian version of the V-RQOL, named “*Qualidade de Vida em Voz – QVV*”, has been proven a valid, reliable and sensitive instrument that specifically assesses patients with voice problems^12^^,^^13^.

Several studies have been carried out to elucidate the relationship between emotional conditions and TMD or dysphonia, but very limited research has been done with individuals presenting both conditions. In addition, the application of quality of life protocols has brought important contributions to the understanding of the impact of the problems presented by patients as well the therapeutic approach in their lives.

The goal of this study was to investigate the correlation between the severity of vocal self-perception and TMD severity, between voice-related quality of life and TMD severity, and between oral health-related quality of life impairment and TMD severity.

## MATERIAL AND METHODS

### Ethical Aspects

The study was approved by the Research Ethics Committee of Bauru School of Dentistry, University of São Paulo (Protocol #173/2007). All women recruited for the study were clearly informed about the research purposes and their acceptance to participate was expressed by their signature on an informed consent form.

### Participants

The study sample was composed by 33 women aged 20 to 40 years (mean age: 25.61 years), with or without dysphonia symptoms, who were recruited at the Bauru campus of the University of São Paulo and the local community.

The following exclusion criteria were used^18^: 1. Two or more teeth missing (excluding third molars); 2. Use of removable denture; 3. Presence of occlusal risk, such as such anterior open bite, unilateral crossbite, overjet greater than 6 mm, and centric relation (CR) slide to an intercuspal position (IP) greater than 5 mm^25^; 4. Presence of severe psychiatric, neurological or motion disorders; 5. Presence of dental pain and unsatisfactory periodontal health; 6. History of head and neck cancer or hormonal problems; 7. Smoking history; 8. Participants who had received or were receiving radiation therapy, those using antidepressants and anticonvulsants, and those who had undergone laryngeal surgery.

### Methods

All selected women were subjected to lifestyle investigation by the application of the Brazilian version of the OHIP-short form (OHIP-14)^21^ questionnaire and the Brazilian version of the V-RQOL protocol (QVV). In addition, their self-perception about the importance of the voice was registered^13^. A questionnaire for determination of the presence and severity of TMD was also used^7^.

### Presence and severity of TMD

The entire sample was requested to fill out a questionnaire for clinical interview containing personal information (name, age, sex, address and telephone number) and questions about symptoms related to the main symptoms of TMD. This questionnaire^7^ was developed based on preexisting questionnaires, and was applied to the women without any examiner interference. The participants answered to 10 questions to assess the severity of their TMD signs and symptoms:

1. Do you feel any difficulty on opening the mouth?
2. Do you feel any difficulty on moving your jaw sideways?
3. Do you feel any discomfort or muscle pain when you chew?
4. Do you frequently have headaches?
5. Do you feel any pain in your neck and/or shoulders?
6. Do you feel any pain inside your ears or next to them?
7. Do you notice any noise in your TMJ?
8. Do you consider your bite “abnormal”?
9. Do you use only one side of your mouth to chew?
10. Do you feel any pain on your face when you wake up?

Three possibilities of answers were offered: “Yes”, “No” or “Sometimes”. Each “Yes” received the value 2, each “Sometimes” received the value 1 and the value 0 was given for each “No” answer. Questions 4, 6 and 7 received value 3 when the answer “Yes” was given to bilateral or intense symptoms, 2 if symptoms were unilateral or soft, 1 for “Sometimes” and zero for the answer “No”^7^. The sum of values allowed classifying the individuals regarding TMD severity as follows: sum of values from 0 to 3: absence of TMD; sum of values from 4 to 8: mild TMD, sum of values from 9 to 14: moderate TMD, sum of values from 15 to 23: severe TMD.

### Quality of life evaluation

Quality of life-related issues were investigated by asking the women to fill out questionnaires at their homes in a condition of privacy. The questionnaires were delivered to the research subjects, who were mostly recruited at the Bauru community. The subjects filled out the questionnaires without interference of the examiner, who was though always available to help them with any doubt.

Both the OHIP-14 and the QVV questionnaires were used to measure the quality of life. The OHIP-14^21^ contains 14 questions that measure the individual’s perception about the impact of their oral conditions on their well-being in the recent months. The results obtained for each question were distributed on a 5-point scoring system (never = 0, almost never = 1, sometimes = 2, almost always = 3 and always = 4). For each of the 7 categories of the questionnaire (functional limitation, physical pain, physical discomfort, physical limitation, psychological limitation, social limitation and incapacity) the mean value assigned for two questions from each categorie was calculated. The final score was calculated by summing the mean values assigned to the questions, totalizing a maximum score of 56 points.

The QVV^13^ is a voice-related quality of life protocol with the purpose of understanding how a voice problem can affect one’s daily activities. It displays a list of possible voice-related issues, and the individuals can respond how their voice was during the last two weeks (1 = excellent, 2 = very good, 3 = good, 4 = reasonable and 5 = bad), representing the vocal self-perception. The questionnaire contains 10 questions that were answered by the participants.

Among the 10 issues, 6 of them (items 1, 2, 3, 6, 7 and 9) cover the physical and functional domain and 4 of them (items 4, 5, 8 and 10) cover the socio-emotional domain. The scale contains 5 response options that correspond to how much each item is considered a problem by the patient, as follows: 1 = it is not a problem, 2 = it is a small problem, 3 = it is a middle/ moderate problem, 4 = it is a major problem, and 5 = it is huge problem. Patients filling out this questionnaire are instructed to answer each question according to the severity of their problem.

According to the authors’ proposed calculations, QVV domains can be calculated separately, using the following equations:

$$\begin{array}{cc} \text{Physical functionality}: & 100-\frac{\left(\text{full score}-6\right)}{24}\times 100 \end{array}$$

$$\begin{array}{cc} \text{Socioemotional}: & 100-\frac{\left(\text{full score}-4\right)}{16}\times 100 \end{array}$$

The full score ranges from 0 (zero) to 100. The higher the value, the better the quality of life. For this calculation, the following equation is used:

$$100-\frac{\left(\text{full score}-10\right)}{40}\times 100$$

### Statistical Analysis

The characterization of the sample was done based on the TMD severity, obtained by the clinical interview questionnaire, through percentage.

For characterization of the quality of life aspects, the OHIP-14 and QVV questionnaires were applied and the mean, median, standard deviation, minimum and maximum values as well as the values of the first and third quartile were used.

Spearman’s Correlation was used to determine the association between TMD presence and severity with the QVV and OHIP-14 results, adopting 5% of significance level.

## RESULTS

The results of the TMD clinical interview questionnaire showed that 8 (24%) participants were considered TMD-free, 15 (46%) had mild TMD, 6 (18%) had moderate TMD and only 4 (12%) were considered as having severe TMD.

The descriptive QVV, OHIP-14 and vocal self-perception measures for the women participating in the study (Table 1) showed a good quality of life. Table 2 presents the results related to the correlation of TMD and the women’s quality of life.

**Table 1:** Descriptive results of the QVV, OHIP-14 and vocal self-perception questionnaires for the women participating in the study

		Mean ± standard deviation	Median	Minimum	Maximum	1^st^ quartile	3rd quartile
OHIP - 14	Functional Limitation	0.17 (± 0.39)	0.00	0.00	1.50	0.00	0.00
	Physical Pain	0.64 (± 0.82)	0.50	0.00	3.00	0.00	1,00
	Psychological Discomfort	0.26 (± 0.56)	0.00	0.00	2.00	0.00	0.00
	Physical Limitation	0.11 (± 0.32)	0.00	0.00	1.50	0.00	0.00
	Psychological Limitation	0.26 (± 0.44)	0.00	0.00	1.50	0.00	0.50
	Social Limitation	0.09 (± 0.32)	0.00	0.00	1.50	0.00	0.00
	Disability	0.09 (± 0.38)	0.00	0.00	2.00	0.00	0.00
	Total	3.21 (± 4.54)	1	0.00	18.00	0.00	5
QVV	Physical	94.68 (± 8.43)	100.00	66.60	100.00	91.60	100.00
	Socio-emotional	98.67 (± 3.04)	100.00	87.50	100.00	100.00	100.00
	Total	96.29 (± 5.97)	100.00	77.50	100.00	92.50	100.00
	Vocal self-perception	2.91 (± 0.77)	3.00	1.00	4.00	3.00	3.00

**Table 2:** Temporomandibular disorders (TMD) presence and severity correlation with the results of QVV, OHIP-14 and vocal self-perception questionnaries

		R	p
TMD X OHIP-14	Functional Limitation	0.18	0.326
	Physical Pain	0.42	0.015 *
	Psychological Disconfort	0.45	0.009 *
	Physical Limitation	0.24	0.187
	Psychological Limitation	0.38	0.030 *
	Social Limitation	0.34	0.053
	Disability	0.41	0.018 *
	Total	0.43	0.012 *
TMD X QVV			
	Physical	-0.45	0.008 *
	Socio-emotional	-0.41	0.017 *
	Total	-0.46	0.007 *
TMD X Vocal self-perception		0.36	0.037 *

R: Correlation values.

* statistically significant at p<0.05.

As shown in Table 2, when the OHIP-14 aspects were associated with TMD, all aspects, except functional limitation (p=0.326) and physical limitation (p=0.187), had statistical significance, and there was a trend to positive correlation between TMD and social limitation (p=0.053). For the rest of aspects analyzed, values of mean positive correlations were detected (0.38 to 0.45 range). In addition, there was negative correlation between the TMD and voice-related quality of life on the total score (p=0.007), physical functioning domain (p=0.008), and socio-emotional domain (p=0.017) as this statistically significant correlation. In this way for this study, the higher the TMD reduced voice-related quality of life and oral health care, as well as the opposite. There were statistically significant positive correlation between TMD and vocal self perception (p=0.037), demonstrating that the higher the TMD severity, the greater self perception of vocal problems by the women analyzed in this study.

## DISCUSSION

TMD is more common in women aged 20 to 40 years, which may be accompanied by muscle-skeletal tension, decrease of voice quality, as well as oral health problems, with an impact on quality of life. However, the findings in literature about the relationship between these aspects are scarce. Thus, in this study two questionnaires of quality of life relating to speech (QVV) and oral health (OHIP-14) were applied to verify if vocal self perception, those related to life and oral health voice present relationship with the severity of TMD.

The study was carried out in a female population because the literature shows a predominance of TMD symptoms in women from 21 to 40 years of age^17^. It has also been shown that the prevalence of TMD is significantly higher among younger women^1^^–^^15^, with a female-to-male ratio of 5:1^1^^–^^8^^,^^10^^–^^15^^,^^21^. In addition, women are more sensitive to not stimuli pain, aged between 24 to 33 years experience changes in their social role, which provokes physical and mental tensions, which could induce the vices of inappropriate mandible use^20^. Another hypothesis is related to an increased occurrence of ligamentary inertia, due to hormonal changes and the level of stress in women^30^. Some authors believe that women are more concerned with health and thus seek treatment more frequently^23^. However, the participants of this study did not sought for treatment related to the TMD.

The application of the TMD questionnaire found the presence of TMD in 25 out of 33 women (76%), and this occurrence was higher than that of a previous study in which TMD was found in 60.63% of the women studied^28^. With regard to TMD severity, the majority of women presented mild TMD (45.45%), supporting the findings of a previous study^29^. Further research might help elucidating the determinants of this occurrence.

Studies that used of OHIP to investigate the quality of life conditions in individuals with TMD^16^^–^^20^^,^^22^^–^^26^ differed from the present study because we used the short version of the questionnaire (OHIP-14). This quality of life protocol has seven dimensions of impact and the results showed statistically significant relationship between TMD and the following aspects: physical pain, psychological discomfort, psychological limitation and disability (p<0.05). These data agree with those of a previous work^25^, which showed a relationship between the presence of pain, as well as the occurrence of two or more TMD problems associated with the quality of life index presented by individuals, and other data where the results of the quality of life are related also to psychosocial axis and the presence of chronic pain^16^.

TMD severity was found to be associated with the presence of changes in the vocal quality^27^. Application of the voice-related quality of life questionnaire and the question about self-perception of vocal problems showed statistically significant results regarding the voice-related quality of life in subjects with TMD, most of them presenting mild TMD.

In addition, vocal self-perception showed positive correlation with the presence of TMD, agreeing with previous data that revealed high incidence of oral disturbances in patients with TMD, including problems concerning speech system^12^, as well as monotone voice quality, hypernasality, hoarse, rough and breathy voice in TMD individuals^4^.

Limitations of the present investigation were not differentiating the various sub-types of TMD and the cross-sectional aspect of the observation. Further studies on this subject are necessary to better understand the exact association between the studied variables.

## CONCLUSION

In the surveyed group of women, there was correlation between TMD and oral health-related quality of life for the following aspects: functional limitation, physical, psychological pain, physical limitation, physical discomfort and disability, as well as on voice-related quality of life questionnaire physical aspects, and socio-emotional, and following with respect to the vocal self perception.
